# Progressive multifocal leukoencephalopathy: the challenge of opportunistic infections in patients with human immunodeficiency virus infection

**DOI:** 10.1590/0037-8682-0506-2022

**Published:** 2022-12-16

**Authors:** Paula Pires da Costa, António José Cruz, João Nuak

**Affiliations:** 1 Hospital do Divino Espírito Santo de Ponta Delgada, Serviço de Medicina Interna, Ponta Delgada, Portugal.; 2 Centro Hospitalar Universitário de São João, Serviço de Doenças Infeciosas, Porto, Portugal.

**FIGURE 1:**
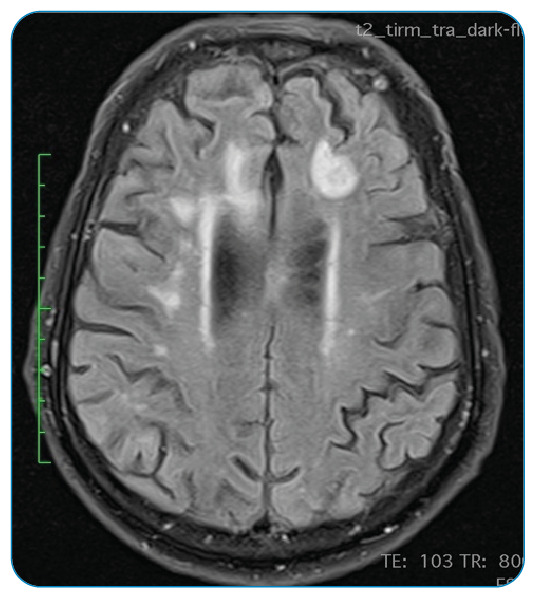
MRI scan on admission. Axial T2-weighted fluid-attenuated inversion recovery. Bilateral frontal subcortical lesional areas with peripheral restriction on difusion and slight vasogenic edema.

**FIGURE 2:**
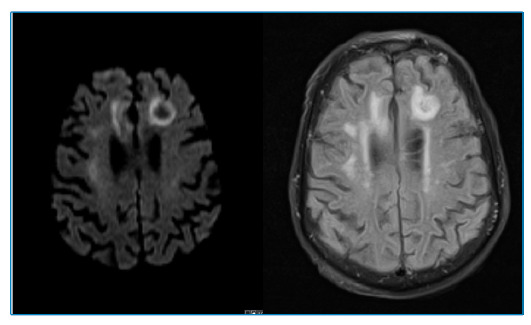
MRI scan 2 weeks after admission. Axial diffusion-weighted imaging and T2-weighted fluid-attenuated inversion recovery showing a persistent peripheral restriction and lack of enhancement with involvement of U-fibers.

A 78-year-old man was admitted to the emergency department because of disorientation, gait imbalance, and dysarthria for 3 weeks. Brain *magnetic resonance imaging (*MRI) on admission revealed frontal subcortical lesions, with hypointense signals in T1-weighted images and hyperintense signals in T2-weighted images, and some round lesions with peripheral restriction of diffusion and slight vasogenic edema (Figure 1). Laboratory tests on admission showed a positive human immunodeficiency virus (HIV) serology, with a viral load of 971,000 copies and a CD4^+^T-lymphocyte count of 111 cells/mm^3^. Under suspected cerebral toxoplasmosis, the patient underwent therapy with sulfadiazine, pyrimethamine, and folinic acid, which was interrupted after 3 weeks considering the lack of clinical improvement and negative polymerase chain reaction for *Toxoplasma* in cerebrospinal fluid with a concomitant negative serum serology. The cerebrospinal fluid analysis also revealed positivity for both John Cunningham (33,000 copies/mL) and Epstein-Barr (5,000 copies/mL) viruses, raising the hypothesis of progressive multifocal leukoencephalopathy (PML) and primary lymphoma of the central nervous system. A new MRI examination showed a persistent lack of enhancement and involvement of U-fibers (Figure 2). Based on all available data, the patient was diagnosed with PML secondary to an initial HIV infection. The patient progressed with clinical worsening with an unfavorable outcome, despite having started combined antiretroviral therapy. PML is a rare, progressive, and often fatal disease that should be included in the differential diagnosis of immunocompromised patients who present with progressive neurological symptoms^1^.The diagnosis is challenging and is usually made based on MRI findings^2^ and high suspicion.
